# Long-term memory in epithelia: transient IFNγ exposure drives stable repression of TFF1 in gastric epithelial cells via epigenetic changes

**DOI:** 10.3389/fimmu.2025.1731220

**Published:** 2026-01-07

**Authors:** Antonia Voli, Daniela Eletto, Fatima Maria Mentucci, Chiara Centrella, Martina Pannetta, Francesco Boccellato, Alfonso Finizio, Silvana Morello, Caterina Giraulo, Amalia Porta, Alessandra Tosco

**Affiliations:** 1 Department of Pharmacy, University of Salerno, Fisciano, Italy; 2 Ph.D. Program in Drug Discovery and Development, University of Salerno, Fisciano, Italy; 3 Ludwig Institute for Cancer Research, Nuffield Department of Clinical Medicine, University of Oxford, Oxford, United Kingdom; 4 Centro Trasfusionale, Ospedale S. Maria della Speranza, Battipaglia, Italy

**Keywords:** gastric mucosoids, *helicobacter*, IFNγ, long-term memory, TFF1

## Abstract

**Introduction:**

Interferon-gamma (IFNγ) is a pro-inflammatory cytokine that is transiently produced and typically activates short-lived JAK–STAT1 signaling, yet it can also induce long-term transcriptional changes. During *Helicobacter pylori* infection, IFNγ persists in the gastric environment, contributing both to host defense and epithelial injury that promotes tumorigenesis. While long-term IFNγ memory has been described in immune cells, its impact on gastric epithelial reprogramming remains unclear.

**Methods:**

We exposed gastric epithelial cells to brief IFNγ stimulation and analyzed gene expression, transcription factor involvement, and epigenetic modifications. Chromatin remodeling at the TFF1 locus was assessed through histone modification analyses, and the role of DNA methylation was evaluated using pharmacological inhibitors. Findings were validated in primary gastric mucosoids exposed to inflammatory mediators released by *H. pylori*-activated immune cells.

**Results:**

Transient IFNγ exposure caused stable repression of TFF1, a gastric tumor suppressor frequently lost in *H. pylori*-associated cancer. This repression persisted after cytokine removal and was mediated by the IFNγ-responsive transcription factor C/EBPβ. Mechanistically, TFF1 silencing was associated with chromatin remodeling, including altered histone H3S10 phosphorylation and H3K9 acetylation at the TFF1 locus. Inhibition of DNA methylation prevented both TFF1 downregulation and C/EBPβ upregulation, indicating that de novo methylation stabilizes the silenced state. Similar durable TFF1 repression was observed in primary gastric mucosoids following exposure to inflammatory mediators.

**Discussion:**

Overall, our findings show that transient inflammatory signals cause durable gene silencing through epigenetic remodeling, revealing how chronic inflammation can reprogram epithelial cells and promote cancer, while suggesting strategies to reverse these effects.

## 1 Introduction

Innate-like lymphocytes (ILLs) provide rapid, early IFNγ production during microbial infection or tissue damage. This rapid response, triggered by cytokines (e.g., Interleukin (IL)-12, IL-18) or direct receptor activation (Pattern Recognition Receptors, antigen receptors), occurs prior to the antigen-specific adaptive immune response.

The binding of Interferon (IFN)γ to its receptor (IFNGR1/2) initiates the canonical Janus kinase/signal transducers and activators of transcription (JAK/STAT) signaling pathway in all studied cells. This cascade involves the phosphorylation of STAT1 by JAK1 and JAK2, followed by STAT1 dimerization, nuclear translocation, and direct gene regulation by binding to GAS (gamma-activated sequence) elements (1).

This canonical signaling is typically transient, peaking within an hour. However, IFNγ also induces sustained gene expression beyond this initial peak. This prolonged response involves indirect mechanisms, such as feedforward loops where IFNγ-induced transcription factors (e.g., IRFs, STATs) amplify the signal (2), and the slow release of IFNγ from a cell-surface phosphatidylserine reservoir (3).

Upon activation, T cells produce IFNγ transiently, typically within a 3–10 hours window (4, 5). Despite this short burst of secretion, IFNγ plays a pivotal role in sustaining long-term host defense against pathogens and tumors, exerting its effects over several days to weeks.

During *Helicobacter pylori* infection, the pro-inflammatory cytokine IFNγ is markedly upregulated, with elevated levels observed in both patients and animal models (6). While IFNγ, secreted mainly by CD4^+^ T helper cells, is essential for fighting *Helicobacter* infections, it can also act as an oncogenic factor by fostering conditions that lead to pre-cancerous changes in the gastric epithelium (7).

We recently demonstrated that within the inflammatory microenvironment induced by *H. pylori* infection, IFNγ drives the silencing of the tumor suppressor gene TFF (Trefoil Family Factor)1 through the transcription factor C/EBPβ (8).

TFF1 is considered a gastro-specific tumor suppressor as *Tff1^KO^* mice spontaneously develop adenomas in the gastric antral/pyloric mucosa, and approximately 30% of these progress to carcinoma (9). Moreover, TFF1 is lost in about 40-60% of GCs for chromosome deletions, somatic mutations, or promoter hypermethylation (10). Interestingly, TFF1 is upregulated during the acute phase of *Helicobacter* infection, likely as a protective response against bacterial aggression (11). However, its expression is progressively silenced as inflammation becomes chronic (12). Consistently, *in vivo* analyses showed *TFF1* downregulation in gastric biopsies from patients with chronic *H. pylori* infection and gastric carcinoma (13).

Epigenetic alterations, particularly DNA methylation and histone deacetylation, are well-established mechanisms of transcriptional repression of tumor suppressor genes in cancer (14). The TFF gene cluster, located on chromosome 21q22.3 and comprising three tandemly arranged genes, contains promoter regions with CpG dinucleotides, although at a lower density than canonical CpG islands. Methylation of these promoters is observed in tissues where TFF genes are not expressed, while unmethylated promoters are associated with active transcription (15, 16).

*Helicobacter pylori* infection is a major risk factor for gastric cancer and is known to induce aberrant promoter methylation in the gastric mucosa. TFF1 is among the genes frequently hypermethylated in this context, with methylation observed in over 50% of *H. pylori*-positive gastric tumors (10, 17). These findings support a model in which chronic infection promotes epigenetic silencing of tumor suppressor genes, thereby contributing to gastric carcinogenesis. However, the upstream signals that lead to TFF1 methylation are not fully understood (18).

Chronic inflammation is a well-known driver of epigenetic dysregulation in cancer. While CpG islands are generally unmethylated in normal cells, persistent inflammatory stimuli, such as those associated with *H. pylori* infection, can lead to *de novo* DNA methylation and the transcriptional repression of tumor suppressor genes (19–21). In this context, the silencing of TFF1 may represent a critical early event in inflammation-driven gastric tumorigenesis.

We hypothesize and aim to determine whether, and through what mechanism, the pro-inflammatory cytokine IFNγ contributes to the long-term silencing of TFF1 in gastric tissues.

In this study, we examine how brief exposure to the inflammatory cytokine IFNγ affects TFF1 expression, with a particular focus on the potential involvement of epigenetic mechanisms.

## 2 Materials and methods

### 2.1 Cell cultures

KATO III cells (gastric carcinoma, derived from a metastatic site and poorly differentiated) were obtained from the American Type Culture Collection (ATCC, Manassas, VA, USA) and maintained in ISCOVE (Euroclone, ECM0192, Italy), supplemented with 20% (v/v) heat inactivated fetal bovine serum (FBS, Euroclone, ECS0180D, South America, origin EU approved) and Penicillin-Streptomycin solution (100 U/mL penicillin and 100 μg/mL streptomycin) (Euroclone, ECB3001D, Italy).

Blood samples were collected from healthy donors (aged 25–40 years) at Centro Trasfusionale, Ospedale S. Maria della Speranza, Battipaglia (Italy) in accordance with the agreement for the use of residual biological materials for research purposes (Prot, n. 0329152, Rep. 783/2023). Human peripheral blood mononuclear cells (PBMCs) were isolated using a previously published protocol (8). Cells were seeded into a petri dish overnight before stimulation.

Gastric mucosoids were generated from organoids using a previously established protocol (22). The organoids themselves were originally established from gastric cells isolated from resected tissue of obese patients at Helios Klinikum (Berlin-Buch, Germany) (**Supplementary Table S1**). Tissue collection was performed with the approval of the Ethics Committee of Charité University Medicine, Berlin (EA1/129/12).

For mucosoid cultures, 200,000 cells derived from antrum organoids were resuspended in 200 μL of culture medium (prepared as described in 22) and seeded onto collagen-coated (Gibco A10644-01, 15 μg/cm²) transwell inserts (Millipore, PIHP01250) placed in 24-well plates.

The space between the filter and the well was filled with 400 μL of culture medium (**Supplementary Table S2**). On day 3 post-seeding, the medium overlying the cells was removed from the well insert to start the Air-Liquid Interface (ALI) culture. Subsequently, the medium below the filter was replaced with 500 μL of medium in which the ROCKi concentration was reduced from 9 µM to 1.8 µM (**Supplementary Table S2**). At this step, only half of the medium volume was replaced regularly to maintain cell-secreted factors in the medium. Foveolar phenotype differentiation was induced by culturing them in a medium lacking WNT3A and R-spondin 1 for one week.

All cells were cultured at 37°C in a humidified incubator with 5% CO_2_.

### 2.2 Bacterial culture conditions and infection experiments

*Helicobacter pylori* P12 strain from a German patient with a duodenal ulcer was cultured on selective Columbia agar (Oxoid, CM0331, Basingstoke, Hampshire, UK) containing 7% (v/v) defibrinated horse blood (ThermoFisher Scientific, SR0050D, The Netherlands) supplemented with an antibiotic mix (DENT, Oxoid, SR0147E, Basingstoke, Hampshire, UK). Bacterial plates were incubated for 3–4 days in a capnophilic atmosphere with 10% CO_2_. Once confluent on the plate, bacteria were scraped using Brain Heart Infusion (BHI, Oxoid, CM1135, Basingstoke, Hampshire, UK) with 10% (v/v) FBS (Euroclone, ECS0180D, South America, origin EU approved) and measured at an optical density at 600 nm (OD_600_), considering 1 OD_600_ = 1 × 10^^8^ bacteria/mL.

To obtain conditioned medium, PBMCs (2 × 10^^6^ cells/well), cultured in 12-well plates, were infected with *H. pylori* at a multiplicity of infection (MOI) of 1:1. The culture supernatant was collected 24 hours post-infection, and IFNγ concentration was measured to assess PBMCs activation, as previously described (8).

### 2.3 Cell treatments

#### 2.3.1 IFNγ stimulation

KATO III cells were seeded in various plate formats depending on the experimental purpose: 12-well plates (3 × 10^^5^ cells per well) for Western blot analysis, 6-well plates (6 × 10^^5^ cells per well) for qPCR, and 100 mm dishes (4 × 10^^5^ cells per dish) for ChIP assays.

For IFNγ stimulation, after 24 hours of seeding, when cells reached 70–80% confluence, they were subjected to a 2-hour pulse of IFNγ (10 ng/mL), followed by washing with PBS and then incubation in complete medium without the cytokine for 24, 48, 72, 96 or 120 hours.

#### 2.3.2 5-AZA treatment

KATO III cells were seeded in 12-well plates (2 × 10^^5^ cells/well) and allowed to grow for 24 hours. Cells were left in starvation overnight without serum and then exposed to 5-AZA (10 μM), also known as Decitabine, a deoxycytidine analogue and DNMT inhibitor, for a total of 72 hours. To maintain a constant drug concentration and counteract its known degradation over time, the 5-AZA–supplemented medium was refreshed every 24 hours. After 48 hours of 5-AZA treatment, cells were subjected to a 2-hour IFNγ (10 ng/mL) pulse.

#### 2.3.3 Infected PBMCs conditioned medium treatment

Three weeks post-seeding, mucosoid cells were cultured for one week in medium lacking WNT3A and R-spondin 1 (-W-R) to induce foveolar differentiation. Subsequently, they were either stimulated for 3 days with conditioned media from non-infected or infected PBMCs, or treated with the conditioned medium for 3 days, followed by another 3 days of incubation in complete medium without the conditioned supernatant.

Twenty-four hours after seeding, KATO III cells were exposed to a 2-hour pulse of conditioned medium from infected PBMCs, followed by washing with PBS and incubation in complete medium without the conditioned supernatant for an additional 72 hours.

### 2.4 RNA extraction and qRT-PCR

Total RNA was extracted using EuroGOLD RNA Pure reagent (Euroclone, S.p.A; EMR506200, Pero, Italy) according to the manufacturer’s instructions and quantified using a NanoDrop 1000 (ThermoFisher Scientific). Its integrity was assessed by electrophoresis on a 1% agarose gel. For cDNA synthesis, 0.2-1 µg of total RNA was reverse-transcribed using M-MLV Reverse Transcriptase (GeneSpin S.r.l., Milan, Italy). Quantitative PCR (qPCR) was performed on a QuantStudio 5 Real-Time PCR System (Thermo Fisher Scientific, MA, USA). Appropriate cDNA dilutions were used for each target gene in a 12 µL reaction volume, using Luna Universal qPCR Master Mix (New England Biolabs, USA). Data from three independent experiments, each performed in technical duplicates, were analyzed using the ΔΔCt method. HPRT1 was used as the endogenous reference gene. Primer sequences are listed in **Supplementary Table S3**.

### 2.5 Western blot analysis

To obtain total protein extracts, cells were resuspended in 1X Laemmli buffer (50 mM Tris-Cl pH 6.8; 2% w/v SDS; 0.1% bromophenol blue; 10% v/v glycerol and 100 mM β-Mercaptoethanol). Mucus from mucosoids was collected and diluted in Laemmli buffer to a final concentration of 2X. Samples were sonicated (1 minute, 10 sec pulse on, 10 sec pulse off, amplitude 28%, Vibra-Cell Sonics). Protein samples were then incubated at 100°C for 5 minutes, centrifuged at 10,000g for 10 minutes to remove cellular debris, loaded into a 10% or 15% polyacrylamide gel according to the proteins molecular weight for electrophoretic separation, and then transferred to an Amersham™ Protran™ Premium 0.45 μm NC (GE Healthcare Life Sciences, GE10600003, Germany) nitrocellulose membrane. Ponceau red staining (0.1% solution in 1% v/v acetic acid) for 5 min was used to evaluate the transfer efficiency. After blocking with 5% w/v of non-fat dry milk (BioRad) for 1 hour or 3% w/v of Bovine Serum Albumine (BSA) (Sigma-Aldrich) for 1 hour the membranes were incubated overnight with primary antibodies at 4°C. After washing three times with TBS-T (Tris-Buffered Saline with 0.05% Tween 20) for 10 min each, the membranes were incubated with secondary antibodies for 1 hour at room temperature, washed three times, and then visualized using a LAS 4000 digital imaging system (GE Healthcare, Waukesha, WI, USA). Band densities were quantified by ImageJ (National Institutes of Health, USA).

A full list of antibodies is provided in **Supplementary Table S4**.

### 2.6 Dual-luciferase reporter assay

For reporter gene assays, KATO III cells (1.5 × 10^5^ cells/well) were seeded in 24-well plates. After 24 hours, cells were transfected using Lipofectamine 2000 reagent (Invitrogen, #11668-027, USA), according to the manufacturer’s protocol. Cells were co-transfected with 1 μg of a pGL3-based plasmid (Promega, #E1751, USA) containing a 1 kb fragment of the TFF1 promoter (from –1036 bp upstream) driving luciferase expression, and 0.1 μg of a plasmid encoding β-galactosidase, used to normalize transfection efficiency. The day after transfection, cells were treated with the DNA methylation inhibitor 5-aza-2’-deoxycytidine (5-AZA, 10 μM) for a total of 24 hours, together with a 2-hour IFNγ (10 ng/ml) pulse.

Luciferase activity was measured using the Dual-Light Luciferase/β-Galactosidase Assay Kit (Applied Biosystems, Foster City, CA, USA). Relative transcriptional activity was expressed as the ratio of firefly luciferase to β-galactosidase activity. All measurements were performed in quadruplicate, and light emission was detected using an EnSpire Alpha Multimode Plate Reader (PerkinElmer, Waltham, MA, USA).

### 2.7 Chromatin immunoprecipitation

ChIP assays were performed using a SimpleChIP^®^ Enzymatic Chromatin IP Kit (Magnetic Beads) (Cell Signaling, 9003, USA) following the manufacturer’s protocol. Briefly, KATO III cells were treated with 1% paraformaldehyde for protein-DNA crosslinking and then with glycine (Cell Signaling, 7005S, USA) for neutralization. Samples were treated with Micrococcal Nuclease (Cell Signaling, 10011, USA) for 20 min at 37°C to obtain DNA fragments of approximately 150–900 bp. Nuclei were sonicated to break the nuclear membrane and chromatin was immunoprecipitated with anti-C/EBP*β* antibody (Cell Signaling, #90081S, USA), anti-H3S10ph antibody (Cell Signaling, 53348, USA), anti-H3K27me3 antibody (Abcam, ab6002) or anti-H3K9ac antibody (Abcam, ab10812) respectively or control IgG overnight at 4°C and then incubated for 2 hours with ChIP-Grade Protein G Magnetic Beads (Cell Signaling, 9006, USA). DNA was eluted, de-crosslinked for 2 hours at 65°C, and purified. The enrichment of C/EBPβ binding or specific histone modifications at the TFF1 gene locus was quantitatively assessed by real-time PCR using the primer sequences listed in **Supplementary Table S5**.

### 2.8 Statistical analysis

Experiments were performed in three independent biological replicates; each measured in technical triplicate or quadruplicate. Results are presented as mean ± standard deviation (SD). Data analysis and graph generation were conducted using Microsoft Excel and GraphPad Prism 4 (GraphPad Software, La Jolla, CA, USA). Figures were assembled using Adobe Illustrator. Statistical significance was assessed using either an unpaired Student’s *t*-test or one-way ANOVA, as appropriate, with a *p*-value ≤ 0.05 considered statistically significant.

## 3 Results

### 3.1 Transient IFNγ exposure leads to sustained TFF1 downregulation

Our recent findings suggest that IFNγ, within the inflammatory microenvironment, drives the silencing of the tumor suppressor gene *TFF1* via the transcription factor C/EBPβ (8). Here, we investigated whether a brief exposure to IFNγ could lead to long-lasting phenotypic changes, with a focus on the sustained repression of TFF1 expression.

To this end, KATO III gastric cancer cells were exposed to 10 ng/mL IFNγ for different pulses (30 minutes or 2 hours), followed by thorough washing and subsequent culture in cytokine-free medium for 24, 48, and 72 hours. As shown in **Supplementary Figure S1A**, both 30 min and 2-hour pulses were sufficient to induce a marked and sustained reduction in TFF1 protein levels, comparable to those observed under continuous IFNγ exposure. Notably, a 2-hour IFNγ pulse reduced TFF1 expression for up to 120 hours after cytokine washout.

**Figure 1:**
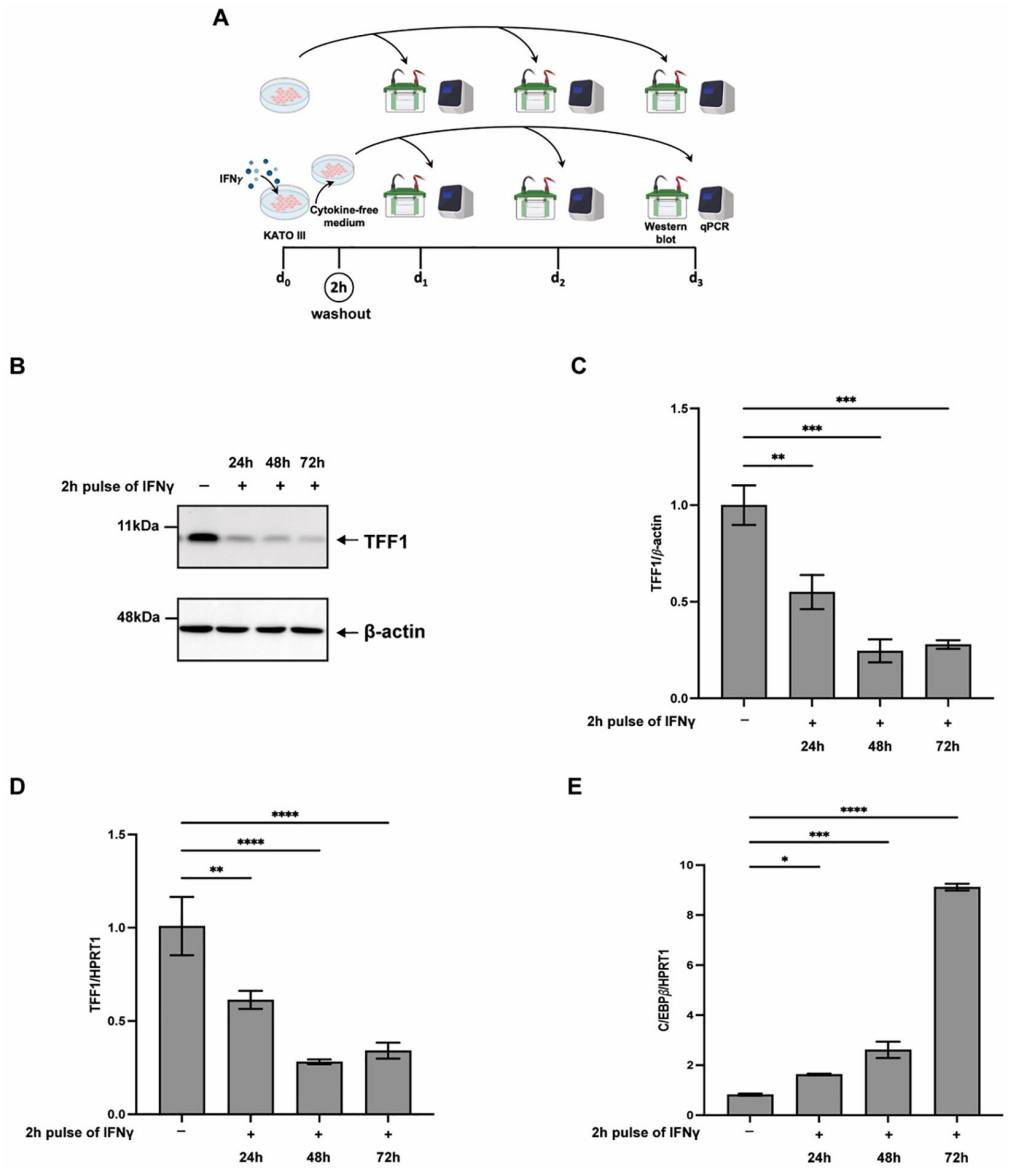
Transient IFNγ exposure downregulates TFF1 and induces C/EBPβ expression in KATO III cells. **(A)** Diagram of the experiment. KATO III cells were pulsed with IFNγ (10 ng/mL) for 2 hours, washed, and then cultured in cytokine free-medium for 24, 48, or 72 hours. **(B)** Western blot analysis of TFF1 protein and **(C)** densitometric quantification of TFF1 protein normalized versus *β*-actin. The image is representative of at least three independent experiments. RT-qPCR analysis of TFF1 **(D)** and C/EBP*β***(E)** expression under the conditions described in **(A)**. HPRT1 was used as the reference gene. Experiments were performed at least in triplicate, and data are expressed as mean ± SD. A multiple comparison test was performed on all data sets after one-way ANOVA to identify significant differences (Tukey’s multiple comparison test; *p ≤ 0.05; **p ≤ 0.01; ***p ≤ 0.001; ****p ≤ 0.0001).

Based on these results, a 2-hour IFNγ pulse with a 72 hours washout was selected for subsequent experiments (**Supplementary Figures S1B, C**). As shown in **Figure 1**, both TFF1 protein (**Figures 1A, B**) and mRNA levels (**Figure 1C**) remained markedly suppressed for up to 72 hours following treatment. In contrast, C/EBPβ transcript levels exhibited a strong and progressive increase, reaching up to a nine-fold induction 72 hours after IFNγ withdrawal (**Figure 1D**). Concomitantly, levels of STAT1 and phosphorylated STAT1 remain elevated even after IFNγ withdrawal (**Supplementary Figure S2**).

These findings indicate that even a brief pulse of IFNγ is sufficient to trigger a transcriptional program resulting in the persistent repression of *TFF1*. This long-lasting effect is likely mediated by sustained activation of transcriptional regulators such as C/EBPβ, despite the absence of ongoing cytokine stimulation.

### 3.2 IFNγ induces long-term epigenetic changes at the TFF1 locus

The prolonged repression of *TFF1* following IFNγ treatment prompted us to explore the potential involvement of epigenetic mechanisms. While only a limited number of studies have examined histone modification dynamics in epithelial cells in response to inflammatory cues, available evidence suggests that chromatin remodeling plays a central role in regulating gene expression under these conditions (23).

Therefore, we examined whether IFNγ suppresses TFF1 gene expression through chromatin-mediated mechanisms. Specifically, we focused on three histone H3 modifications implicated in TFF1 transcriptional regulation in various cellular contexts: phosphorylation at serine 10 (H3S10ph), trimethylation at lysine 27 (H3K27me3), and acetylation at lysine 9 (H3K9ac) (24–27).

Notably, IFNγ has been shown to promote H3K27 trimethylation, contributing to stable gene silencing in macrophages (28). Moreover, Li and colleagues (27) demonstrated that increased levels of H3K9ac and H3S10ph on the TFF1 promoter are associated with transcriptional activation.

**Figure 2:**
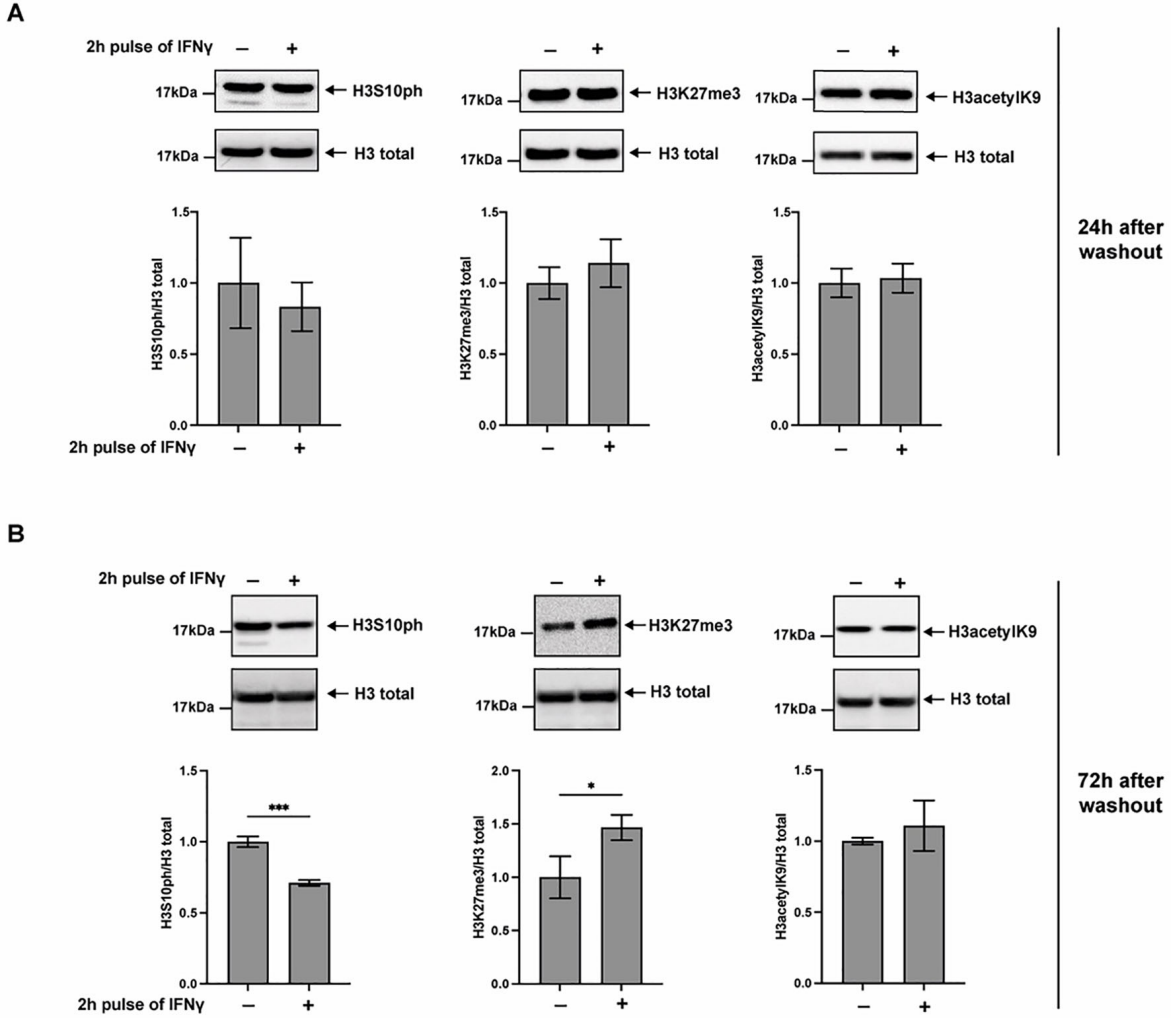
IFN*γ* treatment affects H3S10ph and H3K27me3 levels in KATO III cells. Western blot analysis of histone modifications in KATO III cells treated with IFNγ (10 ng/mL) for 2 hours, followed by incubation in cytokine-free medium for 24 **(A)** or 72 **(B)** hours. Total histone H3 was used as a loading control. Densitometric quantification of H3S10ph and H3K27me3 levels was performed relative to total H3. Experiments were performed in triplicate (*t*-test, *p-value ≤ 0.05, ***p-value ≤ 0.001).

To assess whether IFNγ influences these histone modifications at the global level, we performed Western blot analyses following a 2-hour cytokine pulse and subsequent incubation in fresh medium for 24 or 72 hours. As shown in **Figure 2B**, we observed a significant reduction in H3S10ph and a marked increase in H3K27me3 levels after 72 hours, suggesting a shift toward a more repressive chromatin state. In contrast, no notable changes were observed after 24 hours (**Figure 2A**), indicating that these epigenetic alterations require an extended post-treatment incubation period to become evident. This result suggests that the downregulation of *TFF1* observed during the first 24 hours after cytokine withdrawal is not yet maintained through histone modifications, but is more likely driven by alternative mechanisms, such as the cell-surface catch and release of cytokines mediated by phosphatidylserine (3).

**Figure 3:**
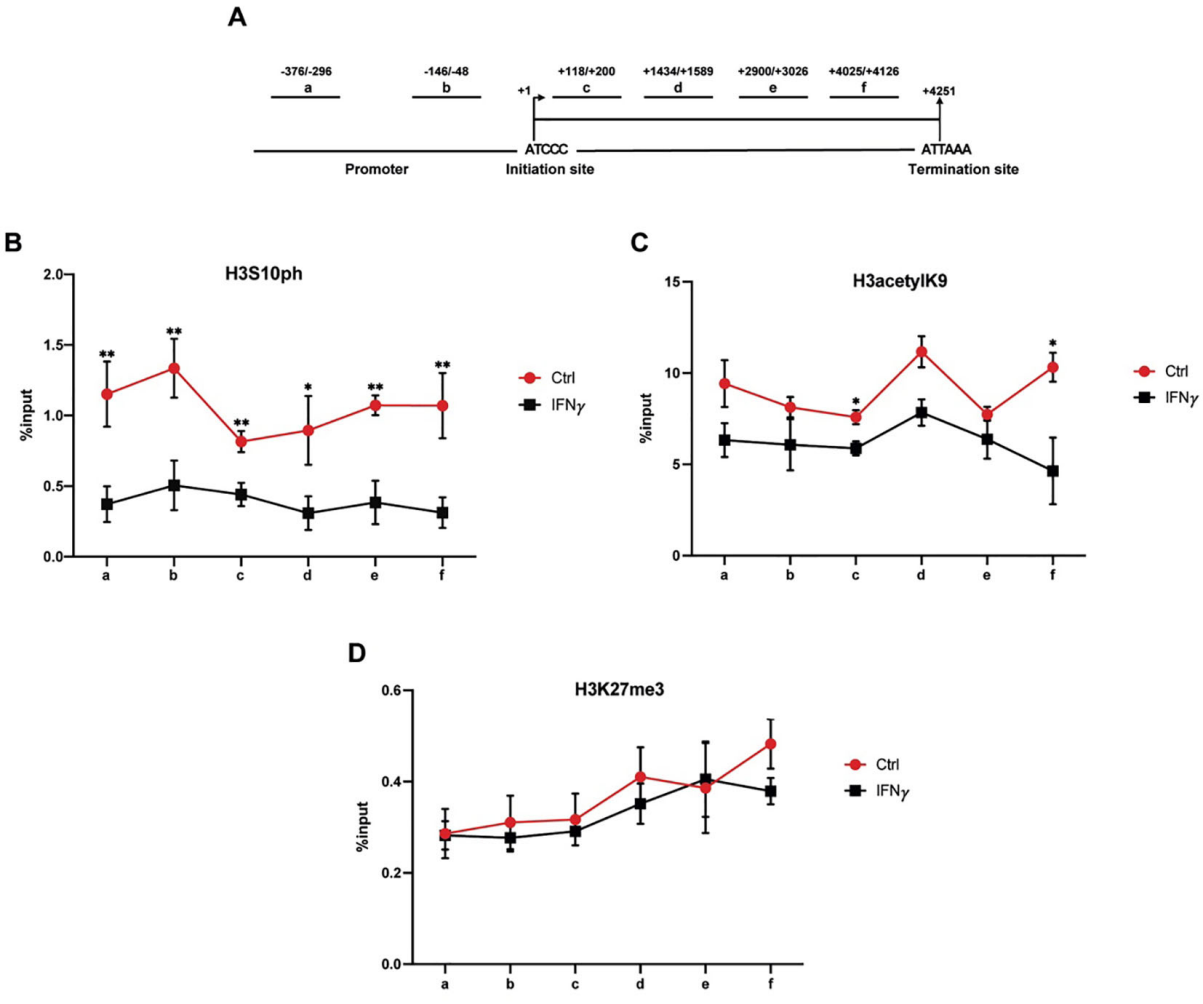
Histone modifications at the TFF1 locus following short-term IFNγ exposure. **(A)** Schematic representation of the ChIP primer sets **(a, b, c, d, e, f)** used to assess histone modifications across the TFF1 promoter and gene body. Quantitative ChIP (qChIP) analysis of H3S10ph **(B)**, H3K9ac **(C)**, and H3K27me3 **(D)** levels in KATO III cells collected 72 hours after a 2-hour IFNγ (10 ng/mL) treatment, followed by culture in cytokine-free medium. ChIP was performed using specific antibodies against the indicated histone modifications. Data represent mean ± SD of triplicate measurements. (*t*-test; *p-value ≤ 0.05, **p-value ≤ 0.01).

Spatial and temporal changes in histone modifications at the TFF1 gene were assessed by chromatin immunoprecipitation (ChIP) in KATO III cells collected 72 hours after a 2-hour IFNγ pulsed treatment. Two sets of primers were designed to target the upstream regulatory region, and one set targeted the first exon of TFF1. Additionally, three primer sets previously described by Li et al. (27), targeting the second and third exons (primers d, e, and f), were included (**Figure 3A**).

Phosphorylation of histone H3 at serine 10 (H3S10ph) is widely associated with transcriptional activation (29). Notably, following the transient cytokine stimulation, a significant reduction in H3S10ph levels was observed both at the TFF1 promoter and within the coding region (**Figure 3B**). These findings are consistent with previous evidence implicating H3S10ph in both transcription initiation and elongation (27). Given that H3S10ph often functions in cooperation with H3K9 acetylation (30), we hypothesized a similar trend for this modification in the context of the TFF1 promoter. Indeed, H3K9ac levels were significantly decreased in the coding region of TFF1, whereas at the promoter region, the reduction did not reach statistical significance (**Figure 3C**). This observation aligns with studies suggesting that H3K9ac, along with other acetylation marks, contributes to chromatin accessibility and may recruit transcriptional co-regulators, particularly during the elongation phase (31).

In contrast, trimethylation of H3K27 (H3K27me3), a mark typically associated with transcriptional repression and stable gene silencing, did not show significant alterations at any region of the TFF1 gene following IFNγ exposure (**Figure 3D**). The apparent discrepancy between the global increase H3K27me3 (**Figure 2**) and the absence of enrichment at the TFF1 locus (**Figure 3D**) suggests that IFNγ promotes a widespread chromatin repressive state, whereas the sustained silencing of TFF1 is driven primarily by specific histone modifications and DNA methylation.

### 3.3 DNA methylation contributes to IFNγ-mediated silencing of TFF1

Given the established role of IFNγ in promoting TFF1 silencing (8), we sought to determine whether cytokine-induced repression of TFF1 in our model also involves alterations in DNA methylation. This hypothesis is supported by evidence linking chronic *Helicobacter pylori* infection to CpG island methylation of tumor suppressor genes (20, 21), as well as by prior findings showing that TFF1 expression is critically regulated by promoter methylation (17).

#### 3.3.1 A short IFNγ pulse is sufficient to trigger methylation-dependent silencing of TFF1

To determine whether DNA methylation contributes to the downregulation of TFF1 following short-term IFNγ exposure, we investigated the effect of inhibiting DNA methylation on cytokine-induced TFF1 silencing.

**Figure 4:**
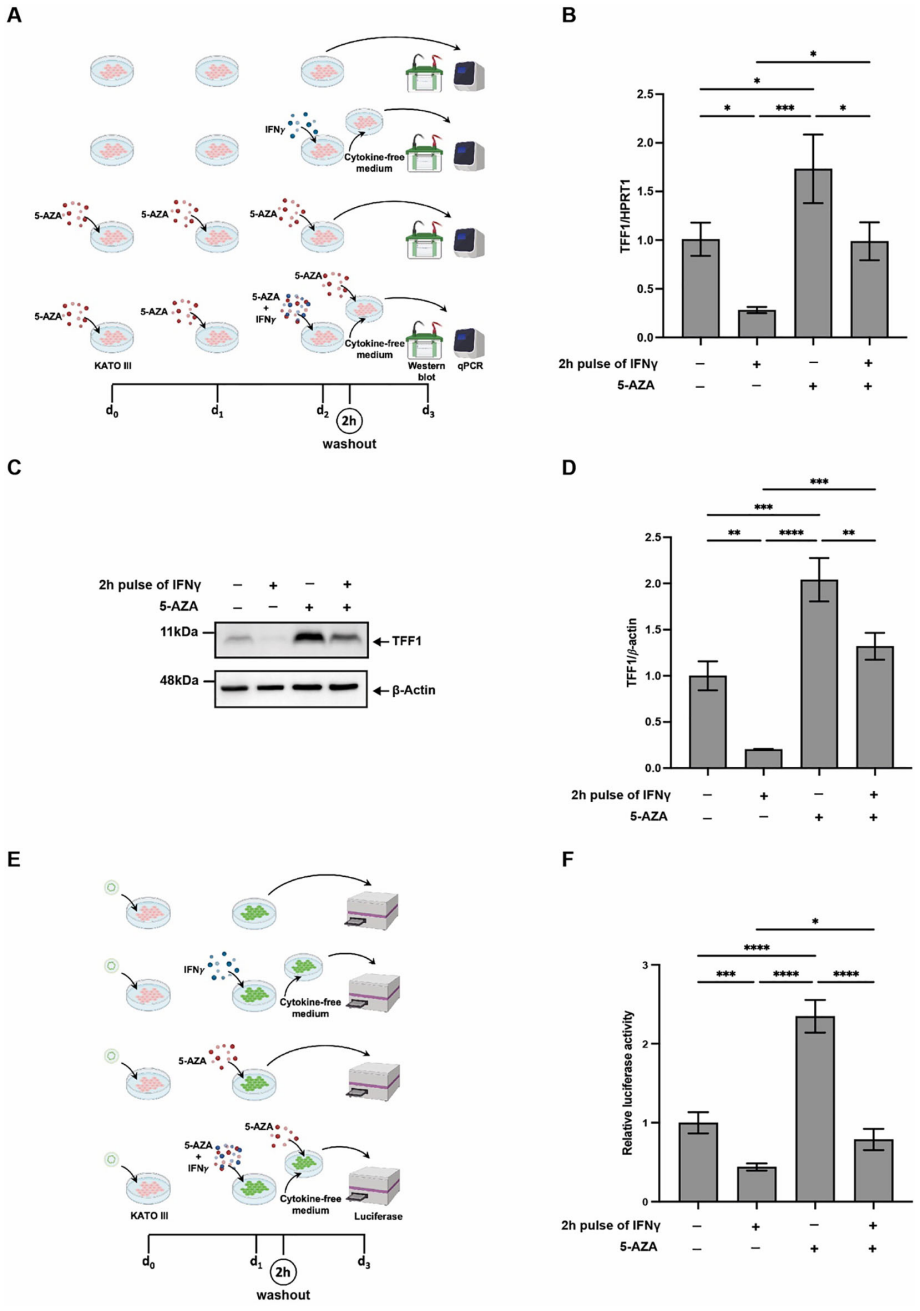
5-AZA prevents the IFNγ-induced TFF1 downregulation induced by short-term IFNγ stimulation. **(A)** Diagram of the experiments in **(B–D)** KATO III cells were treated with the DNA methylation inhibitor 5-aza-2’-deoxycytidine (5-AZA, 10 μM) for a total of 72 hours. After 48 hours of 5-AZA treatment, cells were subjected to a 2-hour IFNγ (10 ng/ml) pulse, followed by culture in cytokine-free medium. **(B)** RT-qPCR analysis of TFF1 mRNA levels in KATO III cells treated as in **(A)** HPRT1 was used as the reference gene. Experiments were performed at least in triplicate, and data are expressed as mean ± SD. **(C)** Western blot analysis of TFF1 protein in KATO III treated as in **(A, D)** Densitometric analysis of TFF1 protein signals normalized versus *β*-actin signals. **(E)** Diagram of luciferase assay in KATO III cells. KATO III cells were transfected with 1.0 kb TFF1 promoter plasmid containing luciferase reporter and after 24 hours cells were treated with the DNA methylation inhibitor 5-aza-2’-deoxycytidine (5-AZA, 10 μM) for a total of 24 hours, together with a 2-hour IFNγ (10 ng/ml) pulse. **(F)** Luciferase assay in KATO III cells. Luciferase reporter activity was normalized versus beta-galactosidase. All data are representative of experiments performed in quadruplicate and are reported as mean ± SD. A multiple comparison test was performed on all data sets after one-way ANOVA to assess if the differences were significant (Tukey’s multiple comparison test, *p ≤ 0.05; **p ≤ 0.01; ***p ≤ 0.001; ****p ≤ 0.0001).

To address this, KATO III cells were treated with the DNA methyltransferase inhibitor 5-AZA, either alone or in combination with IFNγ. Specifically, cells were incubated with 5-AZA (10 μM) for 72 hours, with a 2-hour pulse of IFNγ (10 ng/mL) administered during the final 24 hours, followed by culture in cytokine-free medium (**Figure 4A**).

As shown in **Figure 4B**, inhibition of DNA methylation by 5-AZA led to a significant upregulation of TFF1 mRNA levels. Consistently, intracellular TFF1 protein levels, markedly reduced by IFNγ treatment, were preserved by pre-treatment with 5-AZA (**Figures 4C, D**).

To determine whether this effect was mediated directly through the TFF1 promoter, we performed a luciferase reporter assay using a plasmid (pGL3-1kb-Luc) containing the TFF1 promoter region spanning from –1036 to –17 bp upstream of the transcription start site, following the scheme of **Figure 4E**. KATO III cells transfected with this construct and transiently treated with IFNγ showed a reduced luciferase activity, but when pre-incubated with 5-AZA and then with IFNγ, alone or in combination, this reduction was prevented (**Figure 4F**), suggesting that IFNγ represses TFF1 expression through methylation-sensitive regulatory elements within this promoter region.

These results suggest that even short-term exposure to IFNγ can trigger DNA methylation-dependent mechanisms leading to TFF1 silencing, and that pharmacological inhibition of methylation is sufficient to prevent TFF1 down-regulation under these conditions.

#### 3.3.2 5-AZA suppresses C/EBP*β* induction and modulates promoter binding

Several studies have demonstrated that inflammatory stimuli, including cytokines and bacterial lipopolysaccharide (LPS), induce the transcriptional activation of C/EBPβ by phosphorylation of its transactivation domain, resulting in nuclear accumulation and subsequent binding to consensus binding sites (32, 33). Among these stimuli, IFNγ is known to upregulate C/EBPβ expression (34). In our previous work, we identified C/EBPβ as an IFNγ-responsive gene in KATO III gastric cancer cells and demonstrated that IFNγ stimulation enhances its recruitment to the TFF1 promoter, leading to gene downregulation (8).

**Figure 5:**
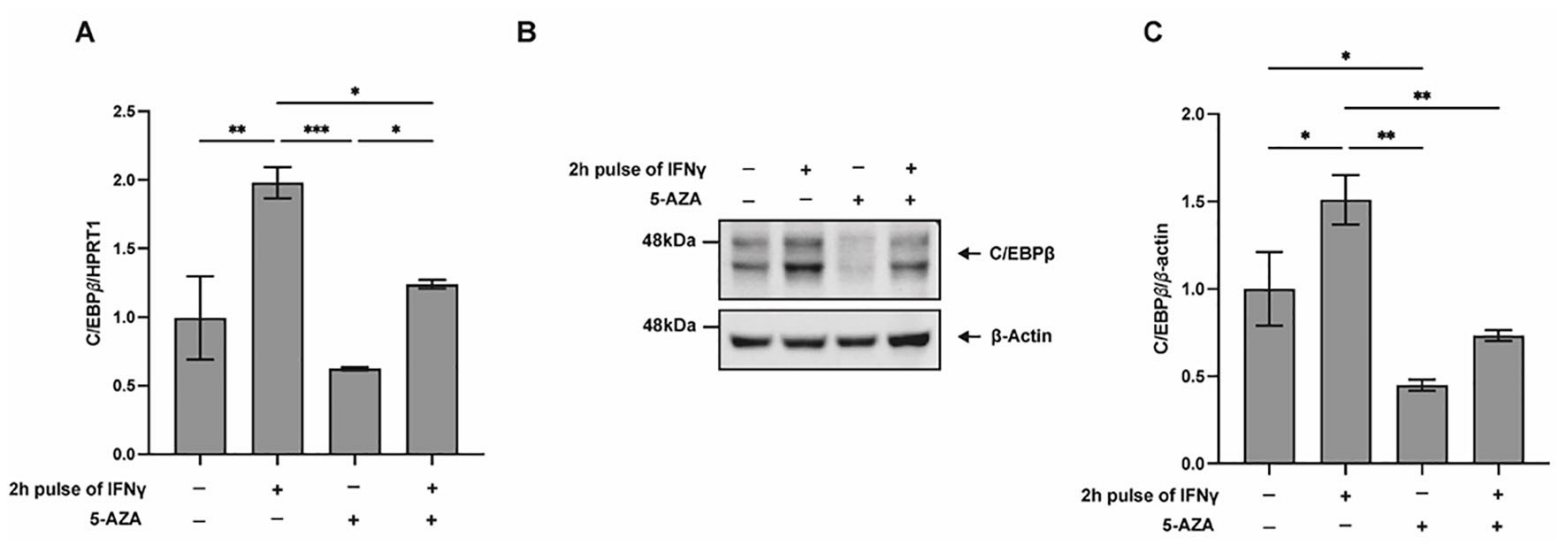
5-AZA blocks IFNγ-induced upregulation of C/EBP*β*. **(A)** RT-qPCR analysis of C/EBP*β* mRNA levels in KATO III cells treated as described in **Figure 4A**. HPRT1 was used as the reference gene. Data represent the mean ± SD from three independent experiments. **(B)** Western blot analysis of C/EBP*β* protein expression in cells treated as in **(A)**. *β*-actin served as the loading control. **(C)** Densitometric quantification of C/EBP*β* protein levels, normalized to *β*-actin. Data are presented as mean ± SD from three independent experiments. A multiple comparison test was performed after one-way ANOVA to assess if the differences were significant (Tukey’s multiple comparison test; *p ≤ 0.05; **p ≤ 0.01; ***p ≤ 0.001.

To investigate whether the combined treatment with 5-AZA and IFNγ modulates C/EBPβ expression and, consequently, its repressive activity on TFF1, we examined whether C/EBPβ transcript and protein levels were altered upon chemical inhibition of DNA methylation following the experimental scheme of **Figure 4A**. As shown in **Figure 5**, 5-AZA treatment significantly attenuated the IFNγ-induced upregulation of C/EBPβ, suggesting a potential attenuation of its repressor function.

**Figure 6:**
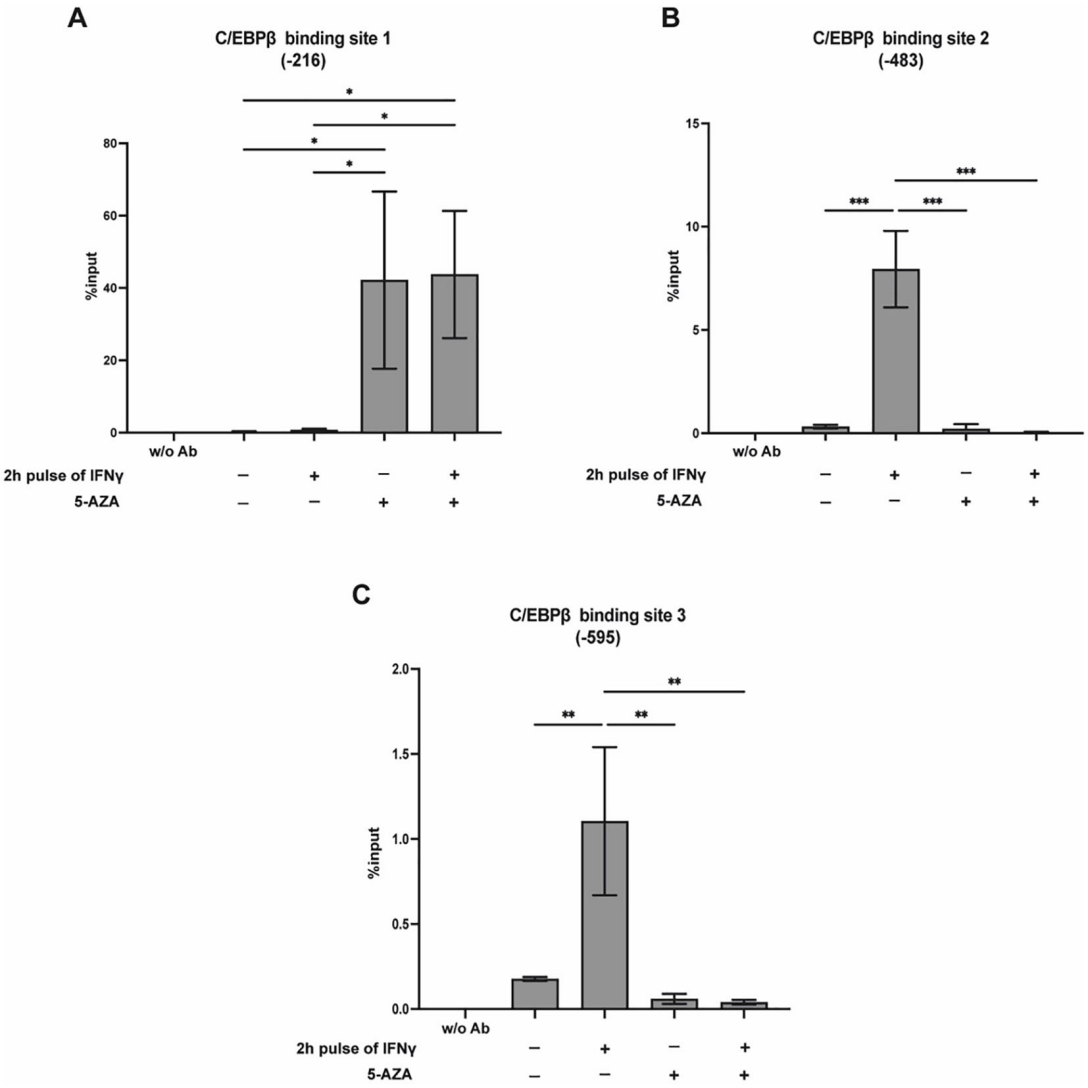
5-AZA modulates C/EBP*β* binding to distinct sites on the TFF1 promoter. ChIP-qPCR analysis of C/EBP*β* binding to the TFF1 promoter in KATO III cells treated as described in **Figure 4A**. The enrichment of C/EBP*β* at binding site 1 (-216) **(A)**, 2 (-483) **(B)**, and 3 (-595) **(C)** of the TFF1 promoter was quantified using qPCR. Results are presented as mean ± SD from three independent experiments. A multiple comparison test was performed after one-way ANOVA to assess if the differences were significant (Tukey’s multiple comparison test, *p ≤ 0.05; **p ≤ 0.01; ***p ≤ 0.001).

C/EBP family members regulate gene expression by binding to specific DNA motifs, many of which contain central CpG dinucleotides. The methylation status of these CpG sites can affect transcription factor binding and subsequent gene regulation. Notably, C/EBP proteins can bind to their recognition sequences with comparable or even enhanced affinity when the CpG site is methylated, both *in vitro* and *in vivo* (35). Of note, three distinct C/EBPβ binding sites are present within the TFF1 promoter (**Supplementary Figure S4**). To examine how DNA methylation affects C/EBPβ binding to the TFF1 promoter in the context of IFNγ stimulation, we performed ChIP-qPCR analysis in KATO III cells treated with IFNγ and/or 5-AZA. The results revealed a differential impact on the three known C/EBPβ binding sites within the TFF1 promoter. Specifically, 5-AZA treatment, particularly in combination with IFNγ, significantly increased C/EBPβ binding to Site 1 (-216) (**Figure 6A**), suggesting that DNA methylation restricts access to this site. Conversely, a distinct trend was observed for Sites 2 (-483) and 3 (-595), where C/EBPβ binding was significantly reduced following combined 5-AZA and IFNγ treatment compared to IFNγ alone (**Figures 6B, C**).

Overall, these results highlight the dual role of DNA methylation in regulating both C/EBPβ expression and its DNA-binding capacity. They also suggest that the methylation status of individual promoter regions can modulate the site-specific recruitment of transcriptional repressors like C/EBPβ during inflammation-induced gene silencing.

### 3.4 *H. pylori*-infected immune cells secrete factors able to suppress TFF1 in gastric primary cells even after their removal

In our study, we observed that TFF1 expression was downregulated in gastric cancer-derived KATO III cells upon exposure to IFNγ alone, whereas in primary human gastric mucosoids, its repression required a combination of TNF-α, IL-1β, and IFNγ (8). These findings highlight how cellular context (transformed versus primary epithelial cells) can influence cytokine responsiveness. They also emphasize the importance of modelling complex inflammatory environments, as exposure to a single cytokine may not be sufficient to replicate the multifaceted signaling that occurs during chronic infection. Our results suggest a central role for the host inflammatory response in silencing TFF1 during the later stages of infection.

Following this hypothesis, we investigated TFF1 expression in mucosoid cultures exposed to conditioned media derived from *H. pylori*-infected immune cells. Specifically, human peripheral blood mononuclear cells (PBMCs) isolated from healthy donors were infected with *H. pylori* at a multiplicity of infection (MOI) of 1:1. The conditioned medium was collected 24 hours post-infection and used to treat both KATO III and human gastric mucosoids.

KATO III cells showed reduced TFF1 expression after a 2-hour pulse of conditioned medium from *H. pylori*-infected PBMCs followed by a 72-hour washout, similar to the effect observed with continuous 72-hour exposure to the conditioned medium. This reduction was also comparable to that seen with IFNγ treatment (**Supplementary Figure S3**).

**Figure 7:**
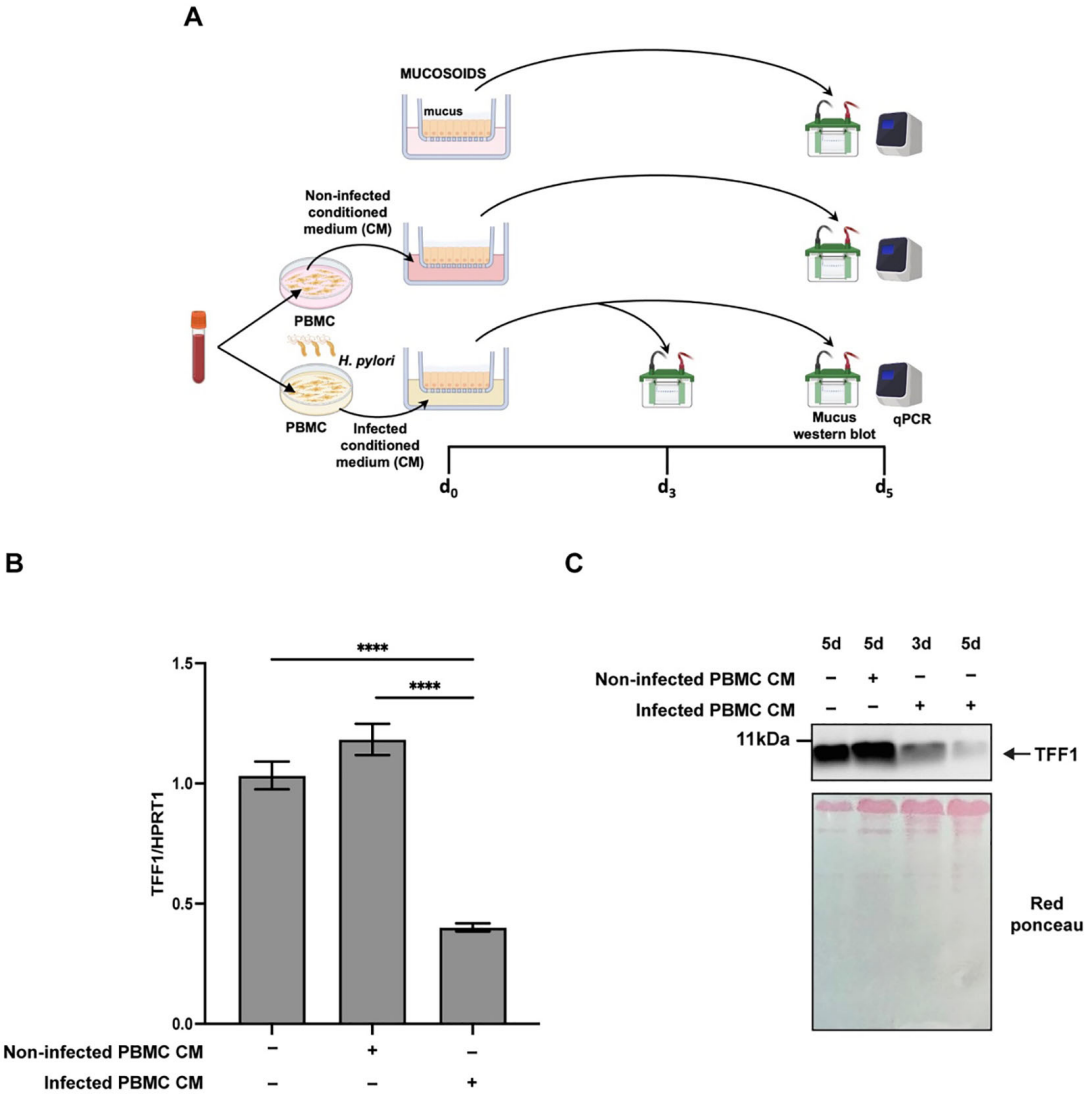
TFF1 expression in mucosoids is affected by inflammatory mediators released by immune cells during *Helicobacter pylori* infection. **(A)** Diagram of the experiments. Mucosoids were treated with conditioned medium from PBMCs infected or not with *H. pylori* (PBMC CM) for 3 or 5 days. **(B)** RT-qPCR analysis of TFF1 expression in mucosoids treated as in A for 3 days. HPRT1 was used as the reference gene. **(C)** Western blot analysis of TFF1 protein secreted in the mucus after exposing mucosoids for 3 or 5 days to the conditioned media (CM) from uninfected PBMCs or infected PBMCs with *H. pylori* at MOI 1:1 for 24 h. Housekeeping proteins are not yet available for the mucus; red ponceau was used as a loading control. A multiple comparison test was performed after one-way ANOVA to assess if the differences in gene expression between different treatments were significant (Tukey’s multiple comparison test, ****p-value ≤ 0.0001).

As shown in **Figure 7**, exposure of mucosoid cells to conditioned medium from *H. pylori*-infected PBMCs (**Figure 7A**) resulted in a marked downregulation of TFF1 expression at 3- and 5-days post-incubation (**Figure 7B**). Consistently, Western blot analysis of the mucus fraction (**Figure 7C**) revealed a significant decrease in secreted TFF1 levels in treated cultures compared to controls.

**Figure 8:**
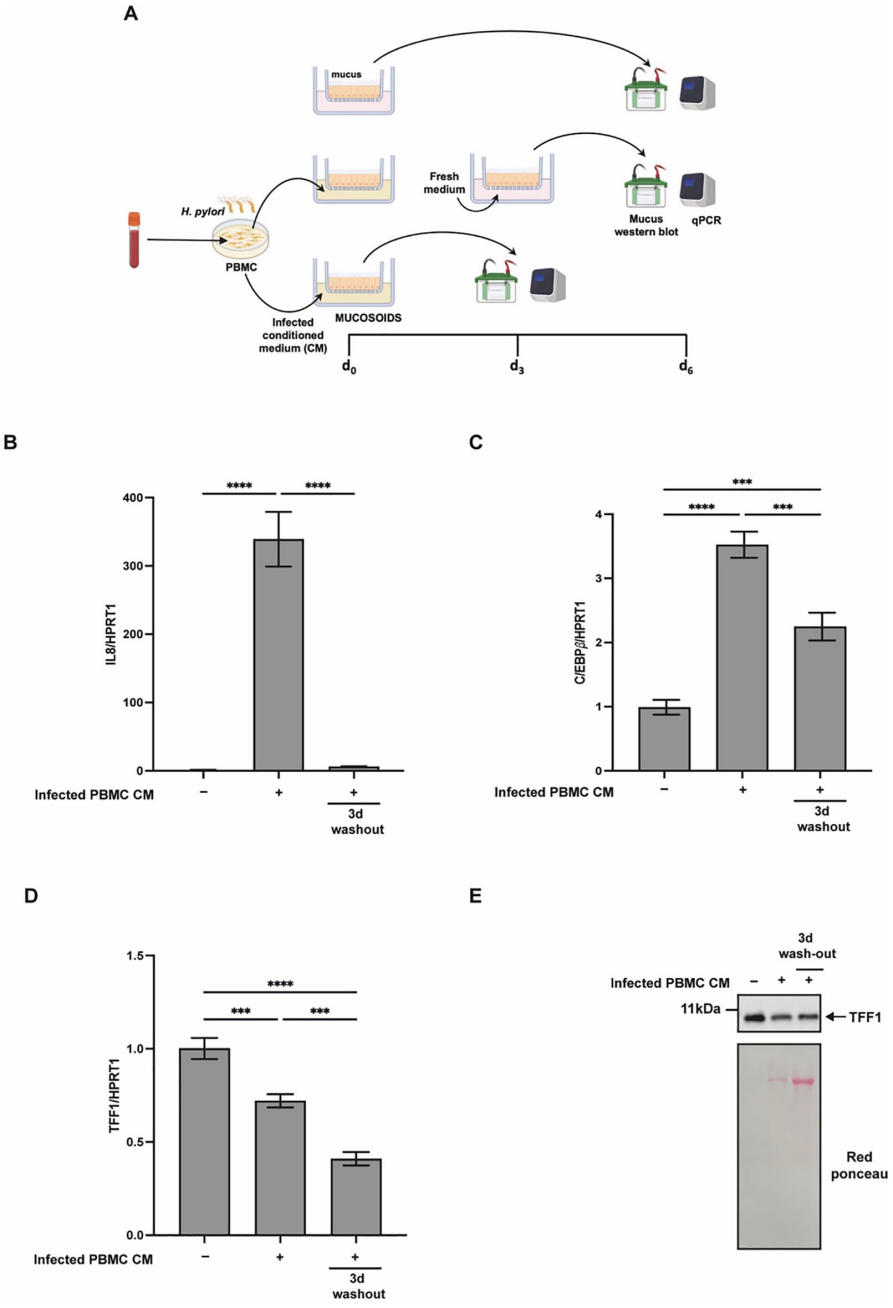
Short-term treatment of mucosoids with conditioned medium from *H*. *pylori*-infected PBMCs is associated with a prolonged downregulation of TFF1 expression. **(A)** Diagram of the experiments. Mucosoids were pulsed with conditioned medium from PBMCs infected with (*H*) *pylori* (PBMC CM) for 72 hours, washed, and cultured in fresh medium for 72 hours. RT-qPCR analysis of IL8 **(B)**, C/EBP*β***(C)** and TFF1 **(D)** expression in mucosoids cells. Gene expression was assessed either immediately after treatment (3 days) or 3 days following a washout period with fresh medium. HPRT1 was used as the reference gene. A multiple comparison test was performed after one-way ANOVA to assess if the differences in gene expression between different treatments were significant (Tukey’s multiple comparison test, ***p-value ≤ 0.001, ****p-value ≤ 0.0001). **(E)** Western blot analysis of TFF1 protein secreted in the mucus after exposing mucosoids as described in **(A)**. Housekeeping proteins are not yet available for the mucus; red ponceau was used as a loading control.

To determine whether the effects of inflammatory mediators persist after their removal, differentiated mucosoids were exposed to conditioned medium from *H. pylori*-infected PBMCs for three days. Following this treatment, the medium was replaced with cytokine-free medium, and the mucosoids were cultured for an additional three days (**Figure 8A**). As shown in **Figure 8B**, IL-8 expression was transiently induced during treatment but returned to baseline after the recovery period. In contrast, TFF1 expression continued to decline even after cytokine withdrawal (**Figures 8D, E**), whereas C/EBPβ remained persistently upregulated (**Figure 8C**). These findings reinforce the notion that, in primary epithelial cells, durable repression of TFF1 requires the complex cytokine milieu produced during infection rather than IFNγ alone.

The results indicate that this unique *in vitro* model (primary human gastric mucosoids incubated with conditioned media from *H. pylori*-infected human immune cells, enriched in pro-inflammatory mediators) provides a more physiologically relevant representation of chronic inflammation. This highlights the importance of considering the combined effects and interactions of multiple factors when examining gene expression changes under inflammatory conditions.

## 4 Discussion

Here, we demonstrate that a transient exposure to pro-inflammatory molecules (mainly IFNγ) induced by *H. pylori* infection can drive the long-lasting silencing of the tumor suppressor gene TFF1 in gastric epithelial cells, connecting transient inflammatory signals to persistent epigenetic changes.

*Helicobacter pylori* infection elicits a strong inflammatory response, largely driven by Th1 cells, whose hallmark cytokine IFNγ plays a central role in antibacterial defense (36). Clinical studies have consistently shown elevated IFNγ levels in the gastric mucosa of *H. pylori*-infected patients compared to uninfected individuals (6), a finding replicated in animal models of infection (37). As a cytokine predominantly secreted by CD4^+^CD25^-^ T helper cells, IFNγ mediates host immune responses contributing to bacterial clearance. However, persistent IFNγ-driven inflammation also promotes tissue damage and chronic gastritis, underscoring its dual role in both protective immunity and disease pathology (7).

*In vivo*, immune cells release cytokines over hours, but an acute immune response typically spans several days. Recent studies have revealed a novel mechanism that sustains IFNγ signaling through cytokine sequestration by phosphatidylserine exposed on the surface of viable cells, followed by its gradual release (3). This reservoir system enables prolonged transcriptional activation and maintains persistent JAK-STAT pathway signaling. Importantly, sustained STAT1 phosphorylation following cytokine withdrawal appears to be the key molecular mechanism driving continued upregulation of antigen presentation genes, thereby ensuring a long-lasting immune response.

However, while STAT1 is essential for the initial priming phase, it is not required to maintain the primed state (38). Instead, transcriptional memory relies on a balance of permissive and repressive chromatin modifications that are differentially inherited in IFNγ-primed cells, thereby preserving the memory of prior IFNγ exposure (39).

Our recent study highlights the role of the inflammatory microenvironment, particularly IFNγ signaling, in driving the silencing of *TFF1*, a well-established tumor suppressor in gastric carcinogenesis, with the transcription factor C/EBPβ playing a key role in this process (8).

Following the previous observations, we investigated the effect of transient exposure to the inflammatory cytokine IFNγ on TFF1 expression. In the KATO III cell system, we observed a stable repression of this epithelial gene, which persisted even 72 hours after cytokine withdrawal. This finding raises important questions about how cytokine-responsive cells convert brief inflammatory signals into long-lasting phenotypic changes. In this context, epigenetic mechanisms emerge as likely mediators, given their ability to produce stable and durable transcriptional effects.

While IFNγ-induced long-term memory is well studied in macrophages, our findings suggest a less investigated function in promoting durable gene repression in epithelial cells. The ability of IFNγ to initiate persistent transcriptional silencing positions it as a potential epigenetic modifier in chronic inflammatory conditions. Here, we show that IFNγ exposure leads to epigenetic repression of TFF1, a gene essential for mucosal integrity (40), providing a mechanistic link between inflammation and epithelial dysfunction.

Our study reveals that transient exposure to the inflammatory cytokine IFNγ can induce stable repression of the epithelial gene TFF1 through coordinated epigenetic remodeling. We demonstrate that this repression is characterized by the loss of active histone marks, the gain of repressive modifications, and additional acquisition of DNA methylation, which together lock TFF1 in a silenced state. These changes are mediated, at least in part, by the transcription factor C/EBPβ, which is upregulated by IFNγ and appears to facilitate the establishment of a repressive chromatin environment. While traditionally viewed as a transcriptional activator in immune and stress responses, C/EBPβ can also function as a repressor depending on cellular context (41). C/EBP family members may help recruit histone deacetylases or polycomb group proteins that promote a repressive chromatin state (42, 43). Notably, C/EBPβ is often upregulated in inflamed or transformed epithelia (41), and its role in facilitating epigenetic silencing suggests it could act as a molecular bridge between inflammatory signaling and epithelial plasticity.

Our chromatin immunoprecipitation experiments reveal that IFNγ treatment causes a reduction of H3S10ph and H3K9ac at the TFF1 gene. These histone changes appear to facilitate the acquisition of DNA methylation, which consolidates the silenced state (44). The repressive histone environment may serve to recruit *de novo* DNA methyltransferases (DNMT3A/B) (45). These findings support a model in which chromatin remodeling and DNA methylation cooperate to establish and maintain stable gene repression. The interplay between histone modifications and DNA methylation contributes to the silencing of target genes such as *TFF1*, thereby reinforcing epigenetic mechanisms of long-term repression.

The observation that 5-AZA treatment prevents TFF1 downregulation in IFNγ-treated cells suggests that DNA methylation is crucial for maintaining the silenced state. Epigenetic therapies such as DNMT inhibitors or HDAC inhibitors could be explored to restore barrier function or tumor suppressor expression in situations of chronic inflammation (46). Since TFF1 loss has been linked to increased epithelial permeability, stemness, and cancer risk (47), reactivating silenced genes may provide a strategy to interrupt inflammation-driven carcinogenesis.

Using conditioned media from *H. pylori*-activated PBMCs, we show that inflammatory signals produced during infection can repress TFF1, also in the mucosoid model of primary gastric cells. This suggests that infection-associated inflammation can serve as an upstream trigger for epigenetic remodeling in epithelial cells. These findings may help explain how chronic infections contribute to the progressive loss of epithelial identity and function in pathogen-associated diseases. The convergence of microbial, immune, and epigenetic signals at key epithelial genes, such as TFF1, underscores the need to study host-microbe interactions through the lens of long-term transcriptional regulation.

Although speculative, these observations open the possibility that epigenetic therapies targeting DNA methylation or histone modifications could restore TFF1 expression and counteract inflammation-driven gastric carcinogenesis.

Collectively, our work provides a mechanistic framework for understanding how transient inflammatory cues can produce long-lasting changes in epithelial identity, with implications for tissue homeostasis, disease progression, and therapeutic intervention.

## Data Availability

The original contributions presented in the study are included in the article/**Supplementary Material**. Further inquiries can be directed to the corresponding authors.
