# Supplementary material for: Long-term memory in epithelia: transient IFNγ exposure drives stable repression of TFF1 in gastric epithelial cells via epigenetic changes
Source: Front Immunol. 2026 Jan 7;16:1731220. doi: 10.3389/fimmu.2025.1731220 (PMC12819787; doi:10.3389/fimmu.2025.1731220)
Supplement: Supplementary file 1 [file DataSheet1.pdf]

Table S1. Weight loss surgery list of patients.

Code	Date of isolation	Age	Gender	Comments
M17A	2015-07-14	30	Female	BMI: 53, *H. pylori* unknown status
M26A	2017-02-17	69	Female	BMI: 50, *H. pylori* negative
M28A	2017-05-16	32	Male	BMI: 43, *H. pylori* negative
M31A	2017-04-12	53	Female	BMI: 58; *H. pylori* negative

Table S2. Composition of mucosoids culture medium.

Name	Concentration	Manufacturer	Code
Advanced DMEM/F-12	18,45% v/v	ThermoFisher, Gibco	12634
Conditioned Wnt3A-medium	50% v/v	Home made	
Conditione R-spondin 1 medium	25% v/v	Home made	
HEPES	1% v/v	Euroclone	ECM0180D
Glutamax	1% v/v	ThermoFisher	35050-087
B27	2% v/v	ThermoFisher	17504044
N2	1% v/v	ThermoFisher	17502048
Human epidermal growth factor (EGF)	20 ng/mL	ThermoFisher	PHG0311
Human noggin	150 ng/mL	ThermoFisher, Prepotech	120-10C-1000
Human fibroblast growth factor (FGF)-10	150 ng/mL	ThermoFisher, Prepotech	100-26-1000
Nicotinamide	10 mM	Sigma	N0636
Human gastrin	10 mM	Sigma	G9145
TGF-β RI Kinase Inhibitor IV	1 μM	Merck	616454-2MG
Y-27632*	9 μM	Merck	Y0503

Note: *after the 3dr day the concentration is reduced to 1,8 μM.

Table S3. Human primers used for qPCR analyses.

Gene		Sequence
HPRT1	Fw	5’-GACCAGTCAACAGGGGACAT-3’
	Rv	5’-CCTGACCAAGGAAAGCAAAG-3’
TFF1	Fw	5’-CCCAGTGTGCAAATAAGGGC-3’
	Rv	5’-TGGAGGGACGTCGATGGTAT-3’
C/EBPβ	Fw	5’-CAAGCACAGCGACGAGTACA-3’
	Rv	5’-AGCTGCTTGAACAAGTTCCG-3’

Table S4. Antibodies used for Western blot analysis.

Antibody	Host	Dilution	Manufacturer	Code
β-actin (C4)	Mouse	1:2000	Santa Cruz	Sc-47778
TFF1	Rabbit	1:500	BioFab	
C/EBPβ	Rabbit	1:1000	Genetex	GTX100675
Phospho-Histone H3 (Ser10) (D7N8E) XP®	Rabbit	1:1000	Cell Signaling	53348
Anti-Histone H3 (tri methyl K27)	Mouse	1:1000	Abcam	Ab6002
Anti-Histone H3 (acetyl K9)	Rabbit	1:1000	Abcam	Ab10812
Anti-Histone H3 (FL-136)	Rabbit	1:1000	Santa Cruz	Sc-10809
Phospho-Stat1 (Ser727)	Rabbit	1:1000	Cell Signaling	9177S
Stat1 (D1K9Y)	Rabbit	1:1000	Cell Signaling	14994S
Anti-Rabbit HRP	Goat	1:2500	Cell Signaling	7074S
Anti-Mouse HRP	Horse	1:2500	Cell Signaling	7076S

Table S5. Primers used for ChIP-qPCR.

Gene		Sequence	Reference
Primer a	Fw	5'-TTGTCACGGCCAAGCCTTTT-3'	
	Rv	5'-TCCCGCCAGGGTAAATACTGT-3'	
Primer b	Fw	5'-TGACCATGTCTAGGAAACACC-3'	
	Rv	5'-AAAGAGCGTTAGATAACATTTGCCT-3'	
Primer c	Fw	5'-CTTTGGAGCAGAGAGGAGGC-3'	
	Rv	5'-CCCCACAGAGCAGGAAGAAG-3'	
Primer d	Fw	5'-TGCGACAAAGACAAAGCG-3'	Li et al., 2011
	Rv	5'-CCGTGGTGAGGGAGGAT-3'	
Primer e	Fw	5'-GAAAGATGCAAAGTCCACAAACC-3'	Li et al., 2011
	Rv	5'-TGTCCAGTGAGGCGGATATAAA-3'	
Primer f	Fw	5'-CCCCTCACCCCTGTAG-3'	Li et al., 2011
	Rv	5'-GCTCTGGGACTAATCACC-3'	
TFF1 (C/EBPβ Binding Site 1)	Fw	5'-GGATTAAGGTCAGGTTGGAGGA-3'	
	Rv	5'-ACGACATGTGGTGAGGTCAT-3'	
TFF1 (C/EBPβ Binding Site 2)	Fw	5'-GTGTTGGGATTACAGGCGTG-3'	
	Rv	5'-AGTGAGAGATGGCCGGAAAA-3'	
TFF1 (C/EBPβ Binding Site 3)	Fw	5'-TGATTCTCCTGACTTAACCTCC-3'	
	Rv	5'-TCACGCCTGTAATCCCAAC-3'	

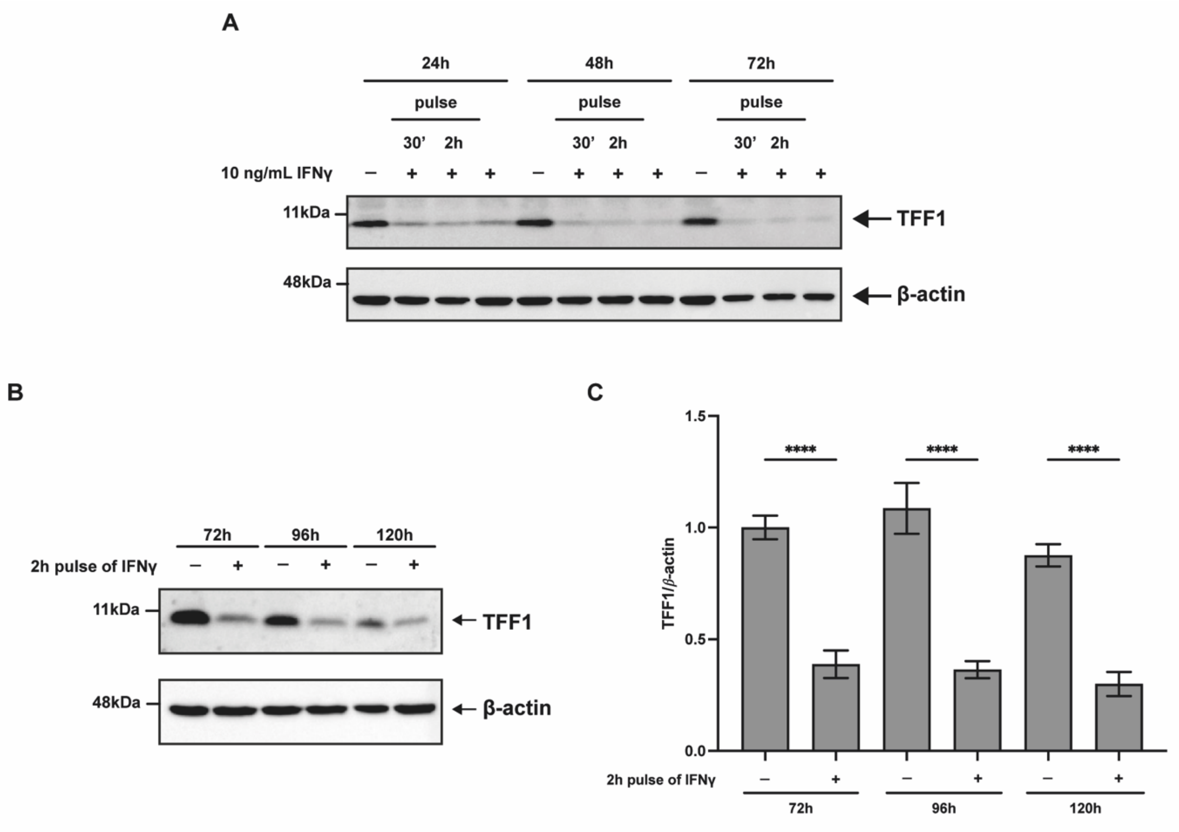

Figure S1. TFF1 expression is significantly reduced up to 120 hours of incubation after a short pulse of IFNγ. (A) Western blot analysis of TFF1 protein levels in KATO III cells exposed to IFNγ (10 ng/mL) either for a 30 minute or 2 hours pulse, or under continuous treatment, and collected after 24, 48, and 72 hours. β-actin served as a loading control. (B) Western blot analysis of TFF1 protein levels in KATO III cells treated with IFNγ (10 ng/mL) for a pulse of 2 hours and collected after 72, 96 and 120 h. (C) Densitometric analysis of TFF1 protein signals normalized versus β-actin signals. All data are representative of experiments performed in triplicate and are reported as mean ± SD. A multiple comparison test was performed on all data sets after one-way ANOVA to assess if the differences were significant (Tukey's multiple comparison test, ****$p \leq 0.0001$).

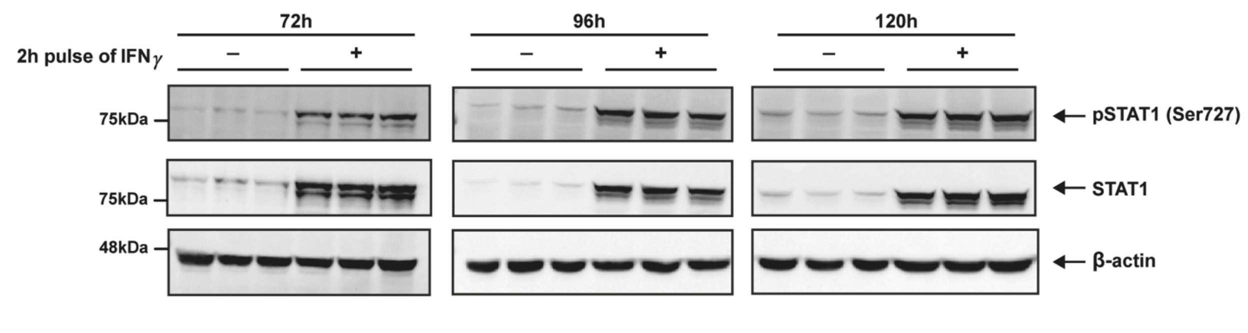

Figure S2. STAT1 expression and phosphorylation are significantly increased up to 120 hours after a short pulse of IFNγ. Western blot analysis of phosphorylated and total STAT1 in KATO III cells treated with a 2 hours pulse of IFN*γ* (10 ng/mL). Cells were collected 72, 96 and 120 hours after treatment. β-actin was included as a loading control. The analysis was performed in triplicate.

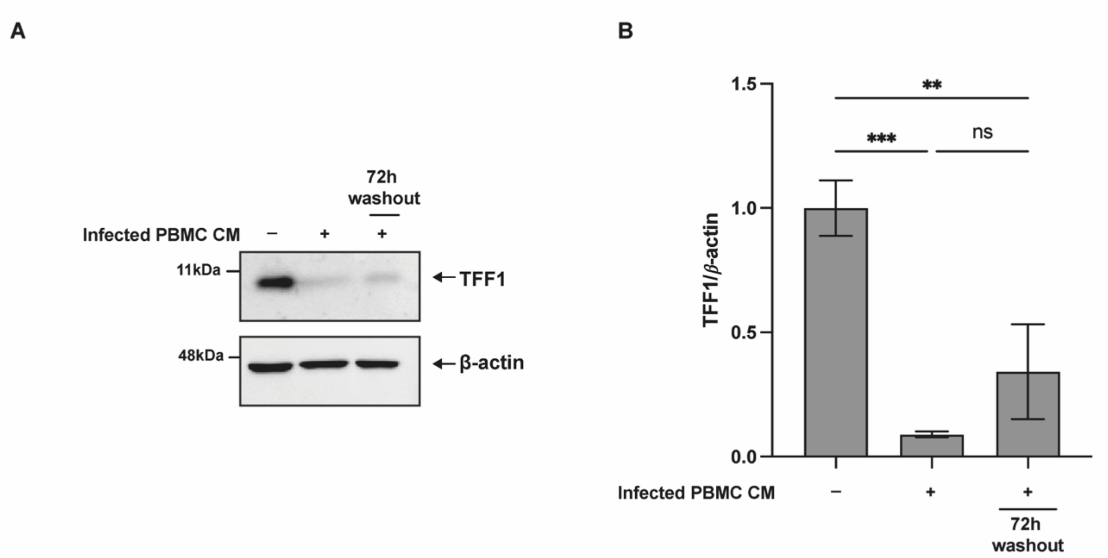

Figure S3. TFF1 expression is significantly reduced up to 72 hours of incubation after a 2 hours exposure to conditioned medium from *H. pylori*-infected PBMC. (A) Western blot analysis of TFF1 protein levels in KATO III cells treated with conditioned medium from PBMCs infected with *H. pylori* (infected PBMC CM) for 72 hours, or pulsed for 2 hours with the same conditioned medium, followed by washing and culture in fresh medium for 72 hours. (B) Densitometric analysis of TFF1 protein signals normalized versus β-actin signals. All data are representative of experiments performed in triplicate and are reported as mean ± SD. A multiple comparison test was performed on all data sets after one-way ANOVA to assess if the differences were significant (Tukey's multiple comparison test, ns=non-significant; ***p* ≤ 0.01; ****p* ≤ 0.001).

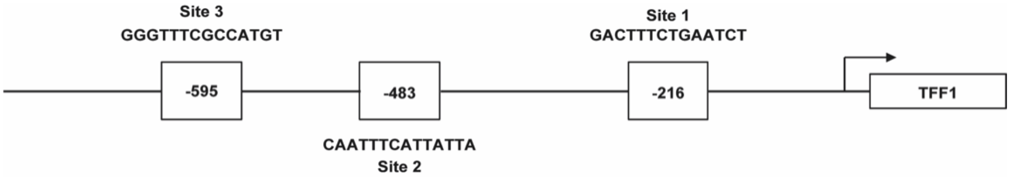

Figure S4. Schematic representation of C/EBPβ binding sites on TFF1 promoter. Site 1 (-216 bp) was previously identified by Sankpal et al., whereas sites 2 (-483 bp) and 3 (-595) are predicted putative binding elements.
